# Complete Genome Sequence of *Pseudomonas protegens* H78, a Plant Growth–Promoting Rhizobacterium

**DOI:** 10.1128/genomeA.00233-17

**Published:** 2017-04-20

**Authors:** Xianqing Huang, Zheng Wang, Yujie Liu, Xuehong Zhang

**Affiliations:** State Key Laboratory of Microbial Metabolism and School of Life Sciences and Biotechnology, Shanghai Jiao Tong University, Shanghai, China

## Abstract

The plant growth–promoting rhizobacterium *Pseudomonas protegens* H78, which was isolated from the rhizosphere of oilseed rape in Shanghai, can produce a large array of antibiotics with a broad spectrum of activities. Here, we report the annotated complete genome sequence of* P. protegens* H78.

## GENOME ANNOUNCEMENT

*Pseudomonas protegens* H78 can produce a set of antibiotics and siderophores, including pyoluteorin (Plt), 2,4-diacetylphloroglucinol (DAPG), pyrrolnitrin (Prn), orfamide, *P. fluorescens* insecticidal toxin, hydrogen cyanide, pyoverdine, and pyochelin (1). The sequencing of the *P. protegens* H78 genome will lay a foundation for further research on the molecular regulation and metabolic engineering of secondary metabolism, including antibiotic biosynthesis in H78.

The H78 genome was determined using the Illumina HiSeq 2000 sequencing platform and the PacBio RSII single-molecule real-time sequencing platform by BGI China. For the Illumina HiSeq 2000 sequencing, two genomic libraries with insert sizes of 0.5 kb and 6.0 kb, respectively, were constructed. A total of 1,058 Mb of clean data (707 Mb and 351 Mb), giving 150-fold coverage of the genome, were assembled into three scaffolds and 14 contigs. In the PacBio RSII sequencing, a total of 132,587 subreads (mean length of 8,363 bp) were *de novo* assembled with SMRT Analysis version 2.3.0. A total of 1,108 Mb of data were produced, giving 158-fold coverage of the genome. Based on these two sets of sequencing data, a complete circular chromosome was successfully assembled.

The complete genome of *P. protegens* H78 contains one circular chromosome of 7,032,394 bp with a G+C content of 63.4%; no plasmid was detected. The H78 complete genome was annotated using Prokka version 1.8 (2). It was predicted to contain 6,446 genes, including 6,290 protein-coding genes, 16 rRNAs, 75 tRNAs, one tmRNA, 17 sRNAs, and 47 miscellaneous RNAs. The H78 genome is homologous to those of *P. protegens* Pf-5 (3), CHA0 (4), and Cab57 (5). The *P. protegens* species can produce a specific spectrum of antibiotic compounds, including Plt, DAPG, and Prn, and has been isolated from the *P. fluorescens* species because of its obvious difference from other fluorescent *Pseudomonas* species (6). However, members of the *P. protegens* species also exhibit a certain degree of strain-specific diversity (5).

### Accession number(s).

The complete genome of *P. protegens* H78 has been deposited in GenBank under the accession number CP013184.
